# In-hospital and mid-term follow-up of low-density lipoprotein cholesterol and target-goal attainment among patients with acute cerebral infarction: a retrospective study

**DOI:** 10.1186/s12944-024-02044-w

**Published:** 2024-02-28

**Authors:** Zhong Chen, Shijia Jin, Yifan Zhang

**Affiliations:** 1 Department of Cardiology, Shanghai Sixth People’s Hospital, Shanghai Jiao Tong University School of Medicine, NO. 600, Yishan Road, Shanghai, 200233 P. R. China; 2 Department of Cardiology, Shanghai Sixth People’s Hospital Fujian, Jinguang Road, Jinjiang, 362200 Fujian P. R. China; 3 Department of Critical Care Medicine, Shanghai Sixth People’s Hospital, Shanghai Jiao Tong University School of Medicine, NO. 600, Yishan Road, Shanghai, 200233 P. R. China

**Keywords:** Atherosclerotic cardiovascular disease, Stroke, Dyslipidemia, Low-density lipoprotein cholesterol

## Abstract

**Objective:**

To investigate the baseline and six-month follow-up data of the main lipid indices as well as low-density lipoprotein cholesterol (LDL-C) target goal attainment in accordance with the current guidelines among patients with acute cerebral infarction (ACI).

**Methods:**

One thousand ninety-nine patients were consecutively enrolled from January 2021 to December 2022 and divided into ACI, old cerebral infarction (OCI), and control groups. General data [sex, age, body mass index (BMI), medications, smoking status, disease history, etc.], baseline data, and six-month follow-up main laboratory data were collected and analyzed. ACI patients were grouped into dyslipidemia and normal groups according to the lipid management guidelines of the European, American, and Chinese populations. Statistical methods were used to screen for possible predictors of dyslipidemia.

**Results:**

Patients with ACI or OCI had higher total cholesterol (TC) and LDL-C levels than did the control group (all *P* < 0.05). According to European (94.7%, 89.0% and 13.4%, *P* < 0.01), American (94.7% vs. 67.7% vs. 45.9%, *P* < 0.001) and Chinese (85.1% vs. 59.1% vs. 18.6%, *P* < 0.001) standards, the proportion of dyslipidemia in the ACI group was greater than that in the OCI and control groups. According to European and American standards, increases in BMI and the estimated glomerular filtration rate (eGFR) are predictors of dyslipidemia in ACI patients. According to Chinese standards, increases in BMI, glycated hemoglobin (HbA1c) levels, and eGFRs are independent predictors of dyslipidemia in ACI patients. The 6-month follow-up of the main lipid levels revealed that among the ACI group, TC, LDL-C and triglyceride(TG) levels (4.86 vs. 3.79, *P* < 0.001; 2.98 vs. 2.01, *P* < 0.001; 1.46 vs. 1.20, *P* < 0.001) and the proportion of dyslipidemia decreased significantly in accordance with European/American and Chinese standards (86.8% vs. 64.6%, *P* = 0.015; 97.2% vs. 84.7%, *P* = 0.012).

**Conclusion:**

These results revealed that lipid management is still not optimal for patients with ACI. More attention should be given to ACI patients with elevated BMI, eGFR, and HbA1c values, which could lead to more individualized lipid management. Although the main lipid levels decreased significantly 6 months after discharge with lipid-lowering therapy, there is still a long way to go to enable more ACI patients to meet the guideline-recommended LDL-C target goal.

**Supplementary Information:**

The online version contains supplementary material available at 10.1186/s12944-024-02044-w.

## Introduction

At present, it was found that the incidence of cerebrovascular disease, which has high mortality and disability rates, continues to increase in China [1] and places a great burden on families and society [2, 3]. In the last decade, low-density lipoprotein cholesterol(LDL-C) management has been widely emphasized in various guidelines [4–6]. LDL-C management in the treatment of cerebrovascular disease has received widespread attention [7–9].

Ischemic stroke is a subgroup of atherosclerotic cardiovascular disease(ASCVD) that can be divided into acute cerebral infarction (ACI) and old cerebral infarction (OCI) depending on the course of the disease. Prevention of cerebrovascular disease is important in clinical practice, and lipid-lowering drugs are currently commonly used [10, 11]. Studies have shown a reduction in the occurrence of ASCVD via a decrease in LDL-C levels [12, 13]. Several studies have further recommended reducing LDL-C levels to very low levels [14]. Therefore, various guidelines have recommended more aggressive management of LDL-C and have set ever-lower LDL-C target goals. This requires more individualized and enhanced lipid-lowering treatment, placing greater demands on clinicians.

Facing such stricter lipid management requirements, there is a lack of investigation into the actual clinical lipid management in China. Therefore, this study was conducted to survey the present situation of In-hospital and mid-term follow-up lipid management among cerebrovascular patients and identify the groups of ACI patients who need enhanced LDL-C management to guide better-individualized lipid management.

## Methods

### Study population

A retrospective study was conducted at Shanghai Sixth People’s Hospital, Shanghai, China, between January 2021 and December 2022 to investigate the baseline and six-month follow-up data of the main lipid indices and LDL-C target goal attainment among patients with ACI and OCI according to the current guidelines. A total of 1099 subjects, including 799 patients with ACI, 128 patients with OCI, and 172 control individuals, were included in the study. Patients who experienced a hemorrhagic conversion from ischemic stroke, coronary heart disease, serious infection, or renal dysfunction[estimated glomerular filtration rate (eGFR) < 30 ml/min/1.73 m^2^] were excluded. Missing or uncertain data on the index test and reference standard were excluded. After enrollment, clinical and biochemical variables were collected. Fasting venous blood was collected to determine hemoglobin levels, white blood cell (WBC) counts, glycated hemoglobin (HbA1c) levels, fasting blood sugar (FBS) levels, blood urea nitrogen(BUN), and creatinine levels. Body mass index (BMI) and the eGFR were calculated by the following formulas.

$${\rm{BMI}} = \,\left( {wight} \right)/\left( {heigh{t^2}} \right)$$

$${\rm{eGFR}}\left( {{\rm{Male}}} \right) = 186 \times {\left( {creatinine} \right)^{ - 1.154}} \times {\left( {age} \right)^{ - 0.203}}$$

$${\rm{eGFR}}\left( {{\rm{Female}}} \right) = 186 \times {\left( {creatinine} \right)^{ - 1.154}} \times {\left( {age} \right)^{ - 0.203}} \times 0.742$$

### Diagnosis of cerebrovascular disease and statin therapy

The diagnosis of cerebrovascular disease, including ACI and OCI, was made by reviewing the clinical features and imaging features of all patients and excluding other systemic stroke-like diseases. Patients with stroke at the acute and subacute stages (within 3 weeks) were defined as having ACI. Patients with a stroke onset ≥ 6 weeks were defined as having OCI [15], while patients with a stroke onset of 3–6 weeks were excluded. Control individuals without ischemic stroke were recruited from Shanghai Sixth People’s Hospital. All participants received moderate-intensity statin therapy (10 mg of rosuvastatin per day/20 mg of atorvastatin per day).

### Quantitative detection of lipid profiles

Fasting venous blood was drawn on the 2nd day after admission and 6 months after discharge. Total cholesterol (TC), triglyceride (TG), high-density lipoprotein cholesterol (HDL-C), and LDL-C levels were determined by the direct method, the catalase clearance assay, with kits purchased from Sichuan Mike Biological Co. via a LABOSPECT 008AS automatic biochemical analyzer. All in-hospital and follow-up indices were uniformly tested by a single central laboratory, and the testers were unaware of the patients’ conditions. Dyslipidemia was classified according to the criteria in the European, American, and Chinese lipid management guidelines (Table S1) [4–6]. All patients were diagnosed by professionally trained clinicians according to diagnostic criteria, and a lipid-lowering treatment was established. All follow-up data were collected by a single professionally trained clinician.

### Statistical methods

Normally distributed continuous variables are given as medians and quartiles. Categorical data are presented as frequencies and percentages. The Kruskal‒Wallis test or chi-square test was used to assess the differences in categorical and continuous variables among the ACI, OCI, and control groups. Logistic regression was used to analyze the risk factors for dyslipidemia in ACI patients. Changes in lipid levels and the proportion of patients with dyslipidemia at admission and at 6 months after discharge were analyzed by the Wilcoxon test and McNemar’s test. The software packages R and SPSS 25.0 were used in this study. All tests were two-tailed, and *P* < 0.05 was considered to indicate statistical significance.

## Results

### Baseline data

As shown in Table 1, 1099 qualified subjects were enrolled in this study. The mean age, height, white blood cell count, hemoglobin level, FBS level, hemoglobin level, HbA1c level, creatinine level, eGFR, smoking status, hypertension and diabetes status and the use of antiplatelet drugs, statins, ezetimibe, mineralocorticoid receptor antagonists (MRAs), angiotensin receptor neprilysin inhibitors (ARNIs), and insulin differed significantly among the three groups.

**Table 1:** Baseline features of patients with ACI, patients with OCI, and control individuals

	ACI(N = 799)	OCI(N = 128)	Control(N = 172)	*P* value
General information				
Age(ys)	69.00(61.00,79.00)	71.50(65.00,79.00)	60.00(53.00,68.00)	< 0.001
Male(n,%)	523(65.5%)	88(68.8%)	98(57.0%)	0.061
Height(cm)	167.00(160.00,172.00)	171.00(158.00,183.50)	167.00(159.00,174.00)	< 0.001
Weight(kg)	65.00(60.00,75.00)	69.00(60.00,76.00)	67.00(59.00,77.00)	0.340
BMI(kg/m^2^)	23.88(22.04,26.12)	24.56(21.54,27.12)	24.19(21.36,27.67)	0.354
Smoking(n,%)	179(22.4%)	54(42.2%)	54(42.2%)	< 0.001
Biochemical indicators				
WBC(*10^9^)	7.10(5.70,8.90)	6.59(5.50,8.25)	6.61(5.55,7.74)	0.003
Hemoglobin(g/L)	138(125,149)	134(120,147)	139(127,155)	0.020
FBS(mmol/L)	5.70(4.83,7.70)	5.20(4.58,6.47)	4.89(4.46,5.63)	< 0.001
HbA1c(%)	6.00(5.60,7.40)	6.20(5.78,6.93)	5.70(5.40,6.40)	< 0.001
BUN(mmol/L)	5.70(4.60,7.10)	5.74(4.92,7.20)	5.63(4.62,6.79)	0.292
Creatinine(.mol/L)	75.15(62.00,90.65)	70.50(61.25,84.75)	70.60(57.20,81.00)	0.001
eGFR[ml/(min*1.73m^2^)]	84.82(69.00,98.07)	86.17(69.30,102.66)	88.37(74.45,107.17)	0.013
History of disease				
Hypertension(n,%)	184(77.0%)	93(72.7%)	78(45.3%)	< 0.001
Diabetes mellitus(n,%)	341(42.7%)	61(47.7%)	44(25.6%)	< 0.001
Medications				
Anti-platelet drugs(n,%)	740(92.6%)	124(96.9%)	172(100.0%)	< 0.001
Statins(n,%)	780(97.6%)	123(96.1%)	160(93.0%)	0.008
Ezetimibe(n,%)	37(4.6%)	46(35.9%)	54(31.6%)	< 0.001
ACEI/ARB(n,%)	310(38.8%)	62(48.4%)	60(34.9%)	0.051
β-receptor blocker(n,%)	160(20.0%)	75(58.6%)	113(65.7%)	< 0.001
MRA(n,%)	24(3.0%)	17(13.3%)	0(0.0%)	< 0.001
ARNI(n,%)	26(3.3%)	11(8.6%)	22(12.8%)	< 0.001
SGLT2i(n,%)	51(6.4%)	13(10.2%)	15(8.7%)	0.215
Insulin(n,%)	250(31.3%)	37(28.9%)	8(4.7%)	< 0.001

ACEI, angiotensin converting enzyme inhibitor; ARB, angiotensin receptor blocker; SGLT2i, sodium-dependent glucose cotransporter inhibitor.

### Main lipid levels, LDL-C attainment, and dyslipidemia among the three study groups

TC, LDL-C, HDL-C, and remnant cholesterol (RC) levels differed significantly among the ACI, OCI, and control groups (all *P* < 0.01) (Table 2). According to Chinese, European, and American standards, the proportion of patients with dyslipidemia in the ACI group was the highest (all *P* < 0.01).

**Table 2:** Lipid levels, LDL-C attainment and dyslipidemia among the three study groups

	ACI(N = 799)	OCI(N = 128)	Control(N = 172)	*P* value
TC(mmol/L)	4.71(3.84,5.56)	3.48(2.91,4.17)	3.84(3.11,4.63)	< 0.001
LDL-C(mmol/L)	2.77(2.06,3.39)	1.89(1.54,2.51)	2.15(1.70,2.81)	< 0.001
TG(mmol/L)	1.28(0.91,1.77)	1.17(0.83,1.57)	1.27(0.92,1.87)	0.090
HDL-C(mmol/L)	1.07(0.92,1.31)	1.01(0.87,1.18)	1.05(0.89,1.25)	0.005
RC(mmol/L)	0.73(0.55,0.98)	0.51(0.36,0.64)	0.52(0.34,0.72)	< 0.001
Dyslipidemia (European Standard)(n,%)	744(94.7%)	113(89.0%)	23(13.4%)	0.003
Dyslipidemia (Chinese Standard)(n,%)	669(85.1%)	75(59.1%)	32(18.6%)	< 0.001
Dyslipidemia (American Standard)(n,%)	744(94.7%)	86(67.7%)	79(45.9%)	< 0.001

### Baseline characteristics of ACI patients grouped into dyslipidemia and normal lipidemia groups

As shown in Table 3, according to Chinese, European, and American standards, ACI patients with dyslipidemia had higher BMIs, HA1c levels, and eGFRs (all P < 0.05).

**Table 3:** Baseline data of ACI patients with and without dyslipidemia

	Dyslipidemia(European/American Standards)(N = 744)	Normal(European/American Standards)(N = 55)	Dyslipidemia(Chinese Standard)(N = 669)	Normal(Chinese Standard)(N = 130)
Age(years)	69(61,79)	72(62,82)	69(61,79)	71(63,80)
Male(n,%)	486(65.3%)	27(64.3%)	444(66.4%)	69(59.0%)
Height(cm)	167(160,172)	166(160,172)	168(160,172)^*^	164(158,170)
Weight(kg)	67(60,75)	62(57,70)	67(60,75)^**^	60(55,70)
BMI(kg/m^2^)	24.03(22.07,26.12)^*^	22.68(20.81,25.54)	24.03(22.22,26.12)^*^	22.89(20.96,25.91)
Smoking(n,%)	167(22.4%)	9(21.4%)	155(23.2%)	21(17.9%)
Hypertension(n,%)	575(77.3%)	30(71.4%)	521(77.9%)	84(71.8%)
Diabetes mellitus(n,%)	318(42.7%)	17(40.5%)	287(42.9%)	48(41.0%)
HbA1c(%)	6.00(5.60,7.50)^*^	5.90(5.40,6.70)	6.10(5.60,7.50)^*^	5.90(5.40,6.63)
eGFR[ml/(min*1.73m^2^)]	84.84(68.90,98.05)^**^	73.52(45.82,88.74)	84.95(70.01,98.09)^*^	78.72(57.04,93.97)

* 0.01 ≤ *P* value < 0.05;** *P* value < 0.01

### Relationship between risk factors and dyslipidemia in the ACI group

**Table 4:** Univariate logistic regression analysis of risk factors for dyslipidemia in the ACI group

	β	SE	Wald	OR(95% CI)	*P* value
European/American Standard					
BMI	0.098	0.048	4.108	1.103(1.003.1.213)	0.043
HbA1c	0.245	0.123	3.974	1.278(1.004.1.626)	0.046
eGFR	0.026	0.006	16.652	1.026(1.013.1.039)	< 0.001
Chinese Standard					
BMI	0.065	0.032	4.114	1.067(1.002.1.136)	0.043
HbA1c	0.169	0.068	6.263	1.184(1.037.1.352)	0.012
eGFR	0.012	0.004	9.804	1.012(1.005.1.020)	0.002

SE: standard error; OR: odds ratio; CI: confidence interval

As shown in Table 4, in the ACI group, elevated BMI, HbA1c, and eGFR values predicted an increased risk of dyslipidemia (European/American Standard) (*P* < 0.05). Moreover, according to the Chinese standard, elevated BMI, HbA1c and eGFR values are also predictors of dyslipidemia (*P* < 0.05).

Further analysis indicated that elevated BMI and eGFR values were independent predictors of dyslipidemia according to the European/American standards (OR values were 1.026 and 1.121, and 95% CIs were (1.012, 1.040) and (1.012, 1.040), respectively; *P* values were 0.021 and < 0.001, respectively). According to the Chinese standard, elevated BMI, HbA1c and eGFR values were independent predictors of dyslipidemia [OR values were 1.070, 1.168, and 1.016; 95% CIs were (1.003, 1.142), (1.009, 1.353), and (1.007, 1.025); and *P* values were 0.039, 0.038, and < 0.001, respectively].

### Changes in main lipid levels and dyslipidemia in patients with ACI 6 months after discharge

**Fig. 1:**
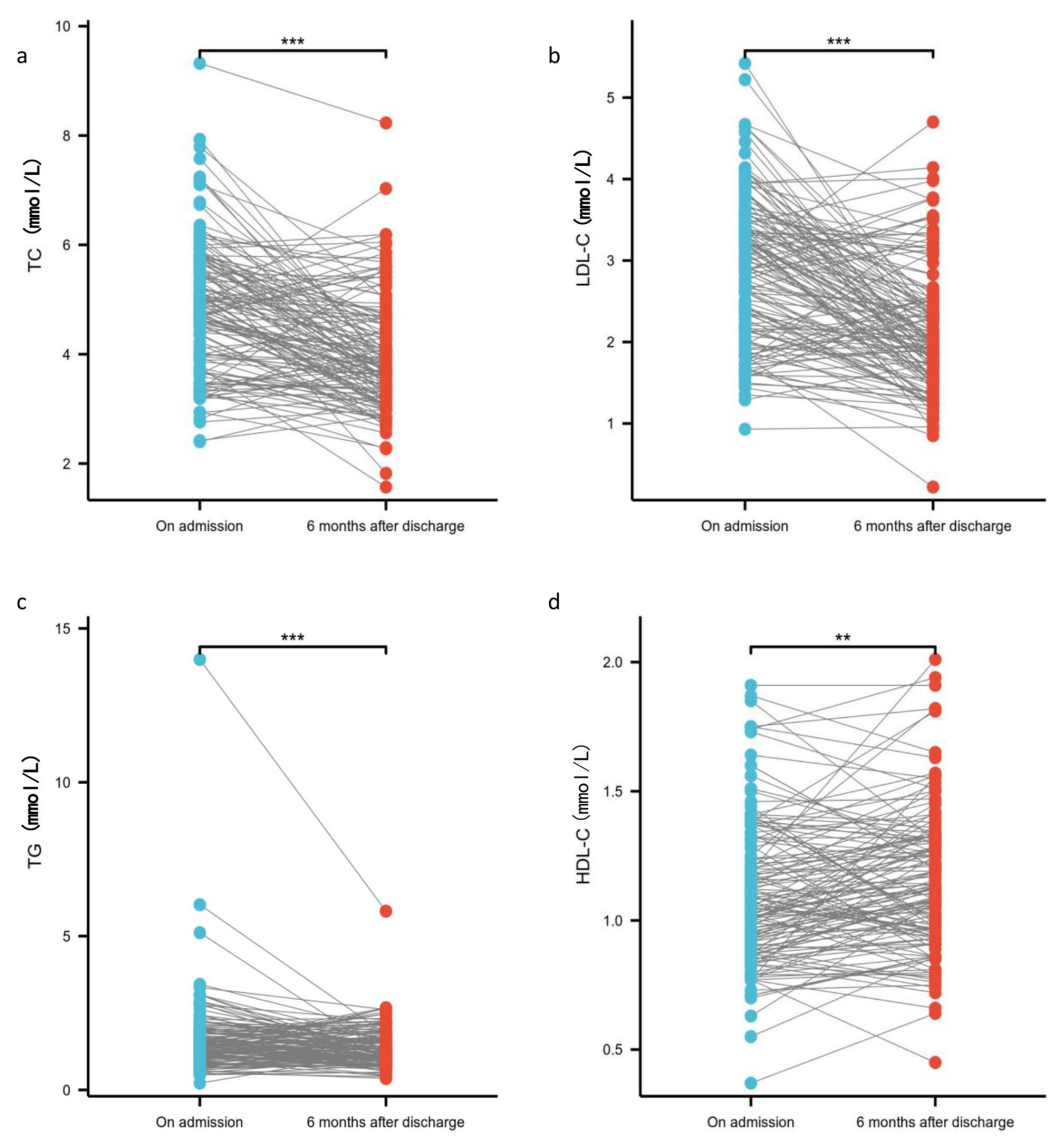
Changes in lipid levels in patients with ACI at admission and six months after discharge. **a**: changes of TC in patients with ACI on admission and six months after discharge; **b**: changes of LDL-C in patients with ACI on admission and six months after discharge; **c**: changes of TG in patients with ACI on admission and six months after discharge; **d**: changes of HDL-C in patients with ACI on admission and six months after discharge. *** *P* value < 0.001;** 0.001 ≤ *P* value < 0.01

**Fig. 2:**
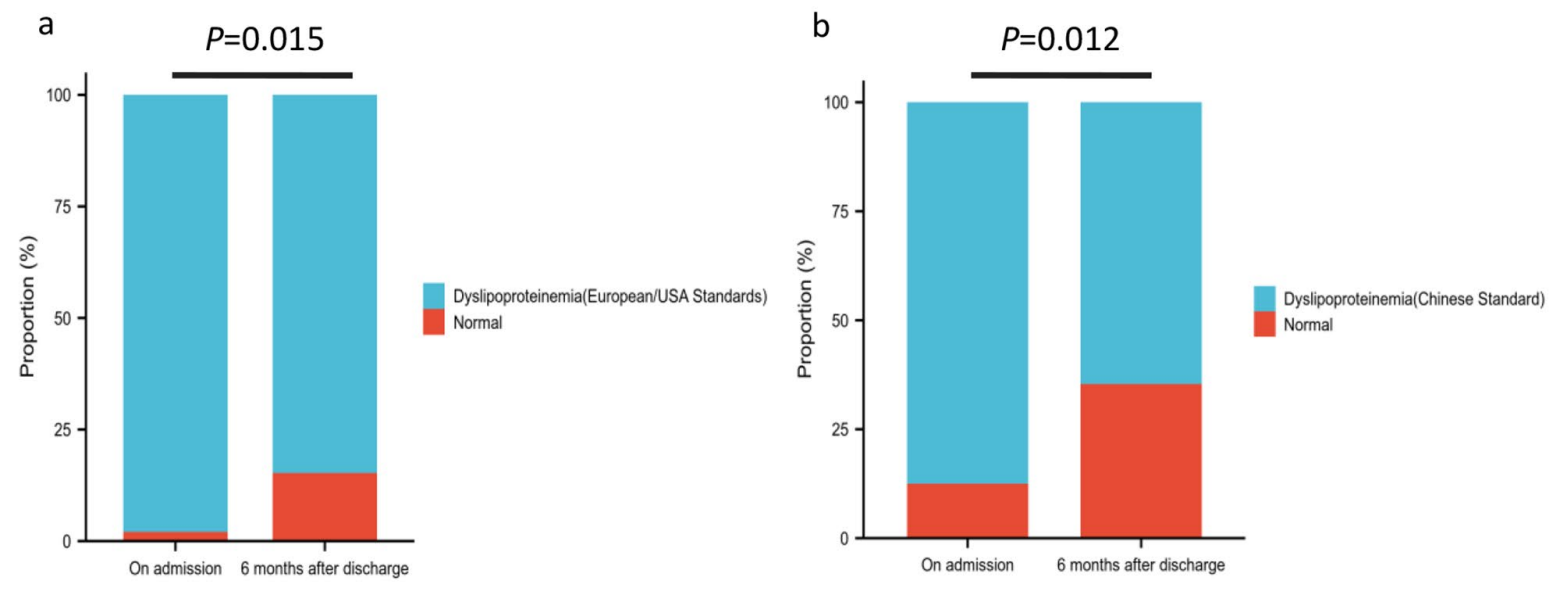
Changes in the proportion of dyslipidemia in patients with ACI at admission and at 6 months after discharge according to different standards. **a**: changes in the proportion of dyslipidemia (European/American standards) in patients with ACI on admission and 6 months after discharge; **b**: changes in the proportion of dyslipidemia (Chinese standard) in patients with ACI on admission and 6 months after discharge

Eighteen percent (144/799) of patients with ACI had their main lipid levels reviewed 6 months after discharge. As shown in Fig. 1, the TC, LDL-C, and TG levels in the ACI patients decreased significantly, while the HDL-C level increased significantly 6 months after discharge (*P* < 0.001, < 0.001, < 0.001 and 0.006, respectively). According to the European, American, and Chinese standards, the incidence of dyslipidemia decreased after 6 months of discharge. However, after 6 months of discharge, according to the Chinese standard, 64.58% of patients (93/144) still did not meet the target-goal level. According to the European and American standards, 84.72% (122/144) of the patients did not reach the target goal level (Fig. 2).

## Discussion

As a critical risk factor for ASCVD [16], LDL-C and its management are now strongly emphasized in the European, American, and Chinese guidelines [17–19]. In the recently updated guidelines, more aggressive lipid management has been recommended, which results in the wide use of lipid-lowering agents. However, the real status of LDL cholesterol control in cerebrovascular patients with such strict lipid management targets is still unclear. This retrospective study showed that LDL-C levels are significantly increased in ACI patients, indicating the necessity of managing LDL-C levels in ACI patients, which was in line with the relevant studies [20, 21]. Moreover, the role of RC in ASCVD has recently received much attention [22, 23]. This work revealed that ACI patients also had greater RC levels. In addition, the significantly higher levels of LDL-C among ACI patients may result from the frequent prescription of lipid-lowering drugs among OCI patients, which leads to lower LDL-C levels. However, most patients with ACI are new-onset patients and have not been regularly treated with lipid-lowering drugs before admission.

Further analysis of the European/American and Chinese standards revealed that LDL-C management was not optimal and that target goal achievement was lower in ACI patients than in healthy individuals, suggesting that aggressive lipid management was still inadequate. Notably, among the 1099 patients in this study, none were given proprotein convertase subtilisin/kexin type 9 inhibitors (PCSK9is), which resulted in lower LDL-C attainment rates. The following reasons may account for the lack of PCSK9i usage: (a) physicians were not sufficiently aware of PCSK9i and did not educate patients well about this drug; (b) the cost of PCSK9i was relatively high, and some patients might refuse to use it due to economic factors; and (c) unlike traditional lipid-lowering drugs, PCSK9i usage requires subcutaneous injections rather than oral administration, which might also impede patients’ willingness to use this medicine.

Further analysis of the predictors of dyslipidemia in ACI patients showed that an elevated BMI was an independent predictor of dyslipidemia according to both the European and American standards and the Chinese standard. It has been widely shown that some dyslipidemia arises from obesity, which is defined as “metabolism-related dyslipidemia“ [24] and is caused by the synergistic effects of insulin resistance, immune cell disorders, adipocyte abnormalities, and vascular dysfunction [25]. Therefore, higher BMI values are usually associated with higher baseline LDL-C levels. Moreover, obesity has been demonstrated to increase the risk of statin intolerance [26], preventing patients with a higher BMI from achieving LDL target-goal levels.

It was also found that higher levels of creatinine could predict a greater risk of dyslipidemia. There is still controversy in relevant studies regarding the variation in LDL-C levels in different renal function groups [27–29]. It was reported by Ho LT et al. that chronic kidney disease (CKD) patients had lower LDL-C levels [29]. As early as 2011, LDL-C level reduction was recommended as the principal therapeutic goal in CKD patients [30]. In the last decade, lipid-lowering therapy for CKD patients has received widespread attention, resulting in better management of lipids in patients with lower eGFRs. In addition, it was found that elevated HbA1c levels were also a predictor of dyslipidemia according to the Chinese standard. The potential reasons might include that (a) elevated HbA1c levels suggest poor glucose management, indicating poor therapy compliance and higher LDL-C levels and that (b) insulin resistance may cause abnormalities in both glucose and lipid metabolism [31]. Taken together, these findings suggest that lipid-lowering strategies for these patients need to be emphasized and individualized.

Furthermore, 6-month data were collected after the discharge of ACI patients, and complete data were available for 144 patients. These data suggest a low rate of follow-up and unoptimistic mid-term post-discharge lipid management for ACI patients, which should be greatly improved. Compared to those at baseline, the TC, TG, LDL-C, and TG levels were significantly decreased, while it was also found that the HDL-C level was increased 6 months after discharge. Moreover, the proportion of patients with dyslipidemia decreased significantly according to both the Chinese and European/American standards, suggesting an apparent improvement in lipid management after professional lipid management interventions. However, a large proportion of patients (75% according to the European/American standards; 60% according to the Chinese standard) still did not achieve the target LDL-C levels, suggesting that more active measures should be taken in terms of aggressive lipid management in patients with ACI.

### Study strengths and limitations

First, this study was conducted in a large general hospital in Shanghai which is the developed city of China. It is a reasonable assumption that the quality of lipid management in this study would be better than the national average, which implies a greater need for improvements in LDL-C target goal attainment nationwide. Second, the lipid management guidelines of several major countries were used for comparison and analysis in this study, allowing a more comprehensive assessment of the real-world status of lipid management.

Several limitations of this study are worth mentioning. (1) This study involved only Chinese patients from a single-center hospital, so the results may be population-specific. (2) Longer-term follow-up data are needed since six months of follow-up is not sufficient. (3) There’s a need for more 6-month follow-up data to clarify the actual status of mid- and long-term lipid management among ACI patients.

## Conclusion

Overall, this study showed that lipid management is still poor in ACI and OCI patients and revealed that more attention should be given to ACI patients with elevated BMI, eGFR, and HbA1c levels, which could lead to more individualized lipid management. Among ACI patients, lipid levels improved significantly 6 months after discharge with lipid-lowering therapy. However, there is still a long way to go to increase patient adherence and enable more patients to maintain lower LDL-C levels and ultimately reduce the burden of ASCVD.

### Electronic supplementary material

Below is the link to the electronic supplementary material.

Supplementary Material 1

## Data Availability

Available by reasonable request.

## Abbreviations

ACEI: Angiotensin-converting enzyme inhibitor
ACI: Acute cerebral infarction
ARB: Angiotensin receptor blocker
ARNI: Angiotensin receptor-neprilysin inhibitor
ASCVD: Atherosclerotic cardiovascular disease
BMI: Body mass index
BUN: Blood urea nitrogen
CI: Confidence interval
CKD: Chronic kidney disease
eGFR: Estimated glomerular filtration rate
FBS: Fasting blood sugar
HbA1c: Glycated hemoglobin
HDL-C: High-density lipoprotein cholesterol
LDL-C: Low-density lipoprotein cholesterol
MRA: Mineralocorticoid receptor antagonist
OCI: Old cerebral infarction
OR: Odd ratio
PCSK9i: Proprotein convertase subtilisin/kexin type 9 inhibitor
RC: Remnant cholesterol
SE: Standard error
SGLT2i: Sodium-dependent glucose cotransporter inhibitor
TC: Total cholesterol
TG: Triglyceride
WBC: White blood cell
