# Supplementary material for: In-hospital and mid-term follow-up of low-density lipoprotein cholesterol and target-goal attainment among patients with acute cerebral infarction: a retrospective study
Source: Lipids Health Dis. 2024 Feb 28;23:62. doi: 10.1186/s12944-024-02044-w (PMC10900700; doi:10.1186/s12944-024-02044-w)
Supplement: Supplementary file 1 — Supplementary Material 1 [file 12944_2024_2044_MOESM1_ESM.docx]

**Table S1** Risk stratification criteria and treatment goals according to the different regional guidelines

|  | European Standard | American Standard | Chinese Standard |
| --- | --- | --- | --- |
| Extreme-risk | - | Progressive ASCVD;  DM, CKD 3/4, or FH patients with ASCVD;  history of premature ASCVD. | - |
| Treatment goal(mmol/L) | - | 1.4 | - |
| Very-high-risk | Documented ASCVD;  DM with target organ damage or early onset of T1DM;  severe CKD;  a calculated SCORE ≥10% for 10-year risk of fatal CVD;  FH with ASCVD or with another major risk factor. | Established or recent hospitalization for ACS, coronary,  carotid or peripheral vascular disease;  10-year risk >20%  diabetes or CKD 3/4 with 1 or more risk factor(s);  FH. | Patients with ASCVD;  DM with target organ damage or with risks including hypertension, smoking, etc.;  severe CKD;  peripheral vascular disease. |
| Treatment goal(mmol/L) | 1.4 | 1.8 | 1.8 |
| High-risk | Markedly elevated single risk factors;  patients with FH without other major risk factors;  patients with DM without target organ damage;  moderate CKD;  a calculated SCORE ≥5% and <10% for 10-year risk of fatal CVD. | ≥2 risk factors and 10-year risk 10-20%;  diabetes or CKD 3/4 with no other risk factors. | Patients with diabetes aged >40 years with 1.3 <LDL-C< 4.9 mmol/L or 3.1 <TC< 7.2 mmol/L;  hypertension patients with ≥2 risk factors;  LDL-C ≥4.9 mmol/L or TC ≥7.2 mmol/L;  moderate CKD. |
| Treatment goal(mmol/L) | 1.8 | 2.6 | 2.6 |
| Moderate-risk | Young patients with DM duration <10 years, without other risk factors; calculated SCORE ≥1% and <5% for 10-year risk of fatal CVD. | ≤2 risk factors and 10-year risk <10%. | With 2-3 risk factors. |
| Treatment goal(mmol/L) | 2.6 | 2.6 | 3.4 |
| Low-risk | Calculated SCORE <1% for 10-year risk of fatal CVD. | 0 risk factors. | With 0-1 risk factor(s). |
| Treatment goal(mmol/L) | 3.0 | 3.4 | 3.4 |

DM, diabetes mellitus; CKD, chronic kidney disease; FH, familial hypercholesterolemia
